# Air-Chambers

**Published:** 1896-07

**Authors:** L. P. Haskell

**Affiliations:** Chicago.


ARTICLE III.

### AIR-CHAMBERS.

DR. L. P. HASKELL, CHICAGO.

Writers still continue to assert the necessity for, and describe the methods of, making air-chambers in full dentures. It seems as though an experience of fifty years, exclusively in prosthetic dentistry, dating back to the first "suction" plates; ought to demonstrate whether air-chambers are a necessity in full dentures.

My preceptor, Dr. Hanson, of Boston, so far as I know, made the first suction plate, I think in the year 1844. The impression was taken in common beeswax; the die made of tin, and the plate fitted to the entire palate. The adhesion was such that he tested its force by soldering a hook to the plate, attached a wire to it, and to this suspended a pail of water, and piling other weights on it, the patient lifted and held the whole. All plates were thus made without air-chambers, which were not introduced till several years later, and were known as the "Gilbert" air-chamber, the same as now used.

For more than twenty-five years I have discarded air-chambers in the full denture as unnecessary, and often very detrimental, in rubber, gold, aluminum, and the heavy continuous-gum work, in flat cases, high arches, ridges hard or soft, and no ridge at all. On my shelves are hundreds of models of every conceivable shape and condition, on which dentures have been made, and all working successfully, and yet not an air chamber in one of them.

There is one fact in connection with the upper jaw that seems to be largely overlooked. The center of the palate in 99 per cent of cases is hard, and is the only portion of the jaw which never changes or yields to pressure. As the alveolus gives way, the plate will rest there and rock, and interfere seriously with its adhesion and stability. In metal plates I make a "relief" by covering the hard center with a thin film of wax, chamfering the margins to a thin edge, or flush with the model.

Now, if there be an air chamber, its anterior and posterior margins must, of necessity, rest on this hard center, and in the course of time the plate is rocking and the air-chamber is worse than useless.

There is a small per cent of cases where the center of the palate is soft, and there is usually a slight crevice. In such cases I make no change at all, but fit the plate closely to the entire surface, making sure that its margin fits snugly into the crevice. On trying the plate in the mouth I do not ask the patient to "suck it up," but am confident that the adhesion will be all right if I see no air-bubbles escape at the rear when pressing the plate with the finger in the center, having previously wet the palatal surface; and this, too, in view of the extra expense involved in making over a continuous-gum set, if the adhesion is not sufficient.

I find many dentists have discarded air-chambers and other appliances for suction; and they are satisfied with the results. These unsightly objects in a metal plate and the unnecessary thickening of a continuous-gum denture can be entirely dispensed with. In rubber sets I bur with the large cone bur a portion of the rubber from the palatal surface.

In the worst case for which I have made a denture in a practice of fifty years, I have no air-chamber. On the right side a portion of the bone was removed on account of disease. The remainder of the ridge is thickened flexible membrane; the center of the palate is enlarged, quite prominent. I raised the model slightly over the right side where it is hard; raised it as usual over the hard center, swaged an aluminium plate, and attached the teeth with rubber. The denture is one inch long in front to restore contour of face. The only favorable condition of the mouth is the retention of all the lower teeth. The patient told me after wearing the plate eighteen months he often forgot he was wearing artificial teeth. Had previously seven sets made by different dentists, none of which had been satisfactory. If an air-chamber is not neec'ed in such a case, where is it a necessity?—*Cosmos.*

